# The lipid nanomicelles incorporating peptide vaccine and SN38 prodrug potentiate neoadjuvant chemo-immunotherapy

**DOI:** 10.1016/j.ijpx.2026.100681

**Published:** 2026-10-09

**Authors:** Jiaxin Guo, Miaoyan Liu, Ping Wan, Yang Zhou, Dexu Luo, Xueheng Wu, Zhiyi Zhou, Xiaowei Huang, Chengzhi Jin, Xin Deng, Jialong Qi, Wenjie Chen

**Affiliations:** a School of Pharmaceutical Sciences, Guangzhou Medical University, Guangzhou, 511436, China; b Yunnan Digestive Endoscopy Clinical Medical Center, Department of Gastroenterology, The First People's Hospital of Yunnan Province, Affiliated Hospital of Kunming University of Science and Technology, Kunming, 650032, China; c State Key Laboratory of Respiratory Diseases, Guangzhou, 511436, China; d Hunan Provincial Key Laboratory of Animal Models and Molecular Medicine, State Key Laboratory of Chemo/Bio-Sensing and Chemometrics, School of Biomedical Sciences, Hunan University, Changsha, Hunan, 410082, China

**Keywords:** Neoadjuvant chemo-immunotherapy, Peptide cancer vaccine, Lipid-based nanomecelles, SN38 prodrug

## Abstract

Neoadjuvant regimens often fail to eliminate micrometastases due to profound immunosuppression and the limited immunogenicity of residual tumor cells. In this work, a versatile lipid nanoplatform was engineered to orchestrate potent chemotherapy with in situ neoantigen vaccination. Unlike conventional physical encapsulation, an arginine-modified SN38 prodrug (SN38-Arg) was synthesized to enhance amphiphilicity and facilitate the co-assembly with PEGylated lipids and tumor-specific peptides (HPV16 E7 or Adpgk). This design yields uniform ~115 nm nanomicelles with exceptional stability and > 60% peptide encapsulation efficiency. Mechanistically, these nanomicelles leverage folate-receptor targeting to achieve dual synchronization: SN38-Arg induces Topoisomerase I inhibition and extensive DNA damage, while simultaneously promoting dendritic cell maturation and antigen cross-presentation. In neoadjuvant murine models of HPV+ TC-1 and MSI-high MC38 tumors, a brief preoperative course of SFE/SFA not only significantly debulked primary tumors but, critically, prevented postsurgical recurrence of treated cohorts and established long-term protective memory T cell reservoirs. This was accompanied by a remodeled tumor immune microenvironment marked by elevated CD8^+^ T cell infiltration and the reduction of regulatory T cells and MDSCs, all without discernible systemic toxicity. This work establishes a clinically translatable, off-the-shelf nanomicellar strategy that integrates SN38-based chemotherapy with peptide vaccination, offering a compelling avenue to improve pathological complete response rates and long-term disease-free survival in surgical oncology.

## Graphical abstract

Graphical abstract of “schematic of SFE/SFA dual mechanisms in tumor models linking immune activation and DNA damage”.

**Figure:**
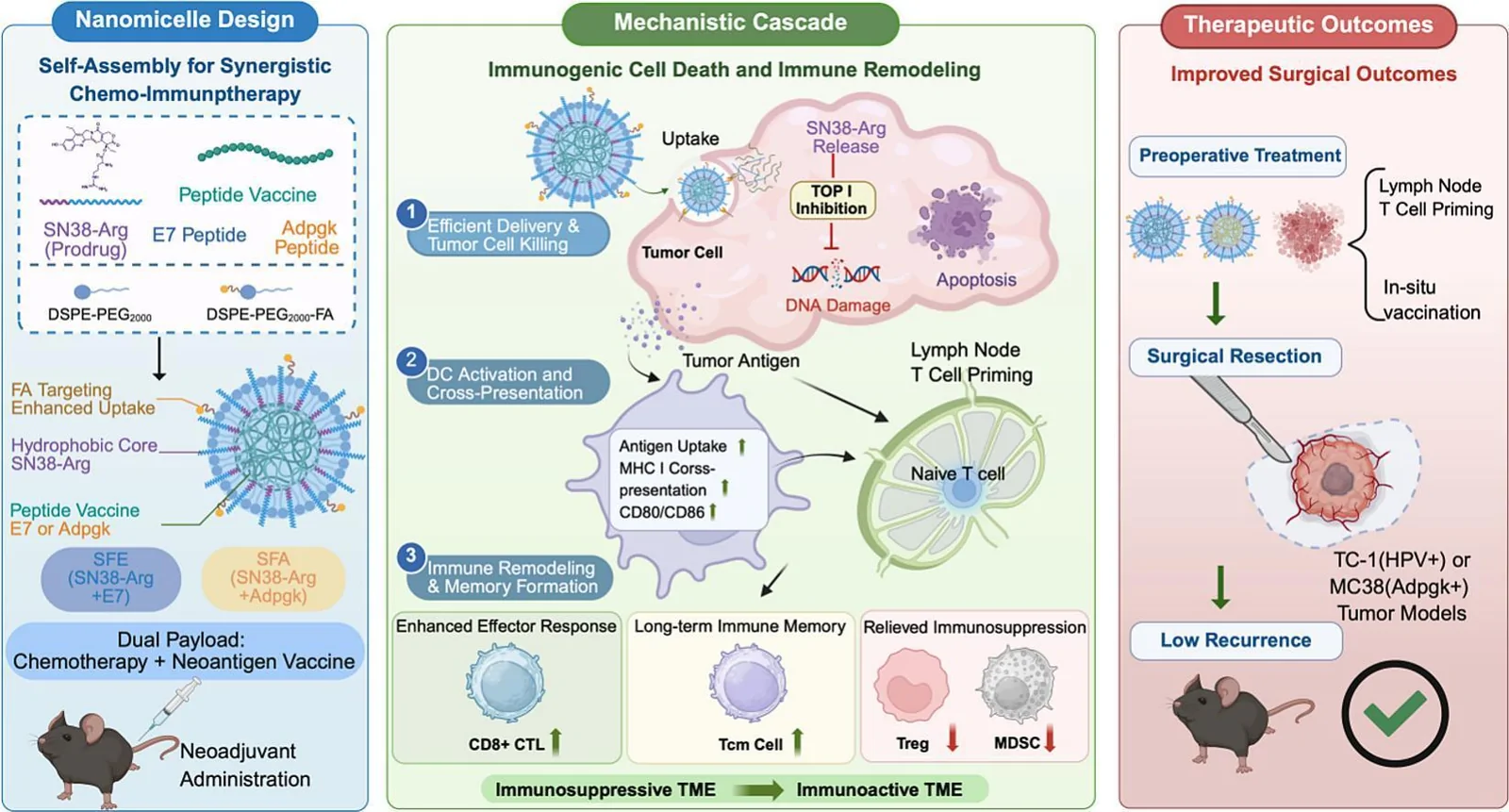

## 1 Introduction

Neoadjuvant therapy, administered prior to definitive surgical resection, constitutes a cornerstone of modern oncology. By reducing primary tumor burden, it aims to facilitate complete (R0) excision and eliminate occult micrometastases, ultimately improving long-term survival (Nair et al., 2023). Despite these theoretical advantages, its clinical translation continues to be challenged by three interrelated obstacles: suboptimal pathological complete response rates, the persistence of an immunosuppressive tumor microenvironment that shields residual tumor cells from immune surveillance, and the high incidence of post-surgical recurrence (Topalian et al., 2023). Therefore, there is a pressing need for neoadjuvant strategies that go beyond cytoreduction to simultaneously establish durable, systemic antitumor immunity capable of eradicating residual disease and preventing metastatic outgrowth. These challenges are exemplified in many cancers including the human papillomavirus (HPV)-associated cervical cancer and colorectal cancer (CRC). HPV infection is a major cause of cervical cancer, although global HPV vaccination programs have reduced cervical cancer incidence, it remains a prevalent gynecological malignancy in China, with persistently high morbidity and mortality rates (Mirabello et al., 2017; Malagón et al., 2024). Patients with advanced or recurrent metastatic cervical cancer face poor prognosis, largely due to the sustained expression of viral oncoproteins such as E7, which drive oncogenesis throughout tumorigenesis (Wang et al., 2024; He et al., 2021; Zhou et al., 2026). However, the relatively small molecular weight and weak antigenicity of E7 peptide may insufficiently activate strong antitumor immune responses (White and Munger, 2017; Cools et al., 2006). While conventional chemotherapy and radiotherapy remain mainstays of treatment but inevitably damage healthy tissues, they often cause significant damage to healthy cells, leading to severe adverse effects (Stoop et al., 2024; Haussmann et al., 2024). Thus, strategies that combine effective tumor debulking with antigen-specific immune activation, while minimizing toxicity, are urgently needed. Similarly, CRC represents one of the most common and lethal malignancies worldwide (Desai et al., 2025; Hu et al., n.d.; Yang et al., 2024a). Despite progress in surgery, radiotherapy, and combination chemotherapy (Chen et al., 2024), patients with locally advanced or metastatic CRC continue to experience unsatisfactory long-term survival, with recurrence and treatment resistance remaining major challenges (Mori et al., 2019; Bradley et al., 2013; Rebollo et al., 2011). In both cancer types, the neoadjuvant window represents an attractive opportunity to deploy combination regimens that couple tumor debulking with antigen-specific immune activation, yet current offerings fail to fully exploit this potential.

Irinotecan (CPT-11), a first-line chemotherapeutic drug for advanced CRC, exerts its efficacy after metabolic activation to SN38, its highly potent metabolite, SN38 demonstrates 10–1000 times greater antitumor activity than irinotecan across various tumor cell lines, underscoring its therapeutic potential (Zhao et al., 2021; Cai et al., 2024; Yang et al., 2024b). However, the direct clinical use of SN38 has been hindered by its extremely poor water solubility, rapid systemic clearance, low tumor specificity, and dose-limiting gastrointestinal toxicity (Mattinzoli et al., 2022; Schumacher et al., 2019). These shortcomings limit its role in neoadjuvant settings for colon cancer and reduce its capacity to induce durable pathological responses. Therefore, innovative strategies to reformulate SN38 for improved solubility, tumor targeting, and immune modulation are critical for expanding its therapeutic utility in colon cancer and beyond. To address these challenges, researchers have employed arginine (Arg) modification strategy. Arginine participates in immune cell proliferation and differentiation through its metabolic conversion to bioactive molecules such as nitric oxide and polyamines (Ishitobi et al., 2022; Canè et al., 2025; Peyraud et al., 2022). Conjugation of SN38 with arginine enhances not only improves amphiphilicity and solubility but also provides immunoregulatory functions by enhancing T cell expansion and functional differentiation, thereby offering a dual chemo-immunomodulatory advantage (Zhang et al., 2022). Nevertheless, SN38-Arg self-assembled micelles along lack sufficient stability for reliable in vivo application. Lipid-based nanomicelle represent a promising strategy for stabilizing SN38-Arg. Amphiphilic lipid components such as DSPE-PEG can encapsulate hydrophobic drugs into stable nanostructures, enhancing solubility, prolonging systemic circulation, and reducing off-target toxicity. For instance, DSPE-PEG_2000_-based paclitaxel nanomicelles demonstrate prolonged circulation half-life via surface PEG layers while significantly reducing allergy incidence compared to conventional paclitaxel formulations (Wang et al., 2019a; Aschmann et al., 2024; Zhang et al., 2024). Building on these advances, we reasoned that co-incorporation of tumor-specific peptide antigens into such lipid-stabilized SN38-Arg nanomicelles would yield an integrated “chemo-vaccine” platform that simultaneously delivers cytotoxic and immunomodulatory payloads.

Furthermore, stimuli-responsive nanomicelles have been engineered to achieve controlled drug release within the tumor microenvironment. For example, PEG2000 conjugated to SN38 through disulfide or carbonate bonds creates a multi-stimuli-responsive prodrug that rapidly releases SN38 upon exposure to GSH, esterase, or H_2_O_2_, achieving vesicle escape and nuclear localization while maintaining SN38-equivalent anticancer activity in vitro (Liu et al., 2017). This integrated design of “stable carrier-targeted delivery-intelligent drug release” effectively compensates for limitations of traditional neoadjuvant chemotherapy. Neoadjuvant therapy, administered prior to definitive surgical resection, aims to reduce tumor burden, facilitate complete excision, and eradicate micrometastatic disease, thereby improving surgical outcomes and long-term survival (Springfeld et al., 2023). Nevertheless, neoadjuvant chemotherapy in the “immune-cold” tumors such as cervical cancer and CRC still faces substantial limitations (Lorusso et al., 2018; Tian et al., 2020; Ling et al., 2016). Although irinotecan and SN38 have been applied in neoadjuvant settings to reduce tumor burden (Zhang et al., 2016; Ando et al., 2024); however, their poor solubility, rapid clearance, and lack of tumor selectivity continue to restrict their effectiveness (Sun et al., n.d.; Xu et al., 2025). Consequently, tumor shrinkage may be achieved, pathological complete response rates remain suboptimal and immunosuppressive microenvironments often persist (Blumenthal et al., 2018).

In the realm of immunotherapy, neoantigen-based peptide immunotherapy offers a complementary strategy (Peng et al., 2019; Yamamoto et al., 2019; Yang et al., 2025). Neoantigens are tumor-specific peptides derived from somatic mutations that can bypass central tolerance and elicit highly specific T lymphocyte activation, memory formation, and durable antitumor immunity (Schumacher et al., 2019; Gupta et al., 2021). However, peptide vaccines alone are prone to enzymatic degradation and inefficient uptake by antigen-presenting cells, resulting in less immunogenicity and suboptimal efficacy. The synergy between SN38-based chemotherapy and peptide vaccination may offer a particularly appealing solution in a neoadjuvant context: SN38 induces chemotherapy that exposes tumor antigenic epitopes, while co-delivered peptide vaccines amplify neoantigen-specific T cell priming and memory, thereby converting the primary tumor into an in-situ vaccine depot (Zhang et al., 2023; Aaes and Vandenabeele, 2021).

In this study, we designed a versatile, lipid-based self-assembled nanomicelle platform that co-loads SN38-Arg and tumor-relevant peptide vaccines for neoadjuvant chemo-immunotherapy. For cervical cancer, folate-modified nanomicelles were engineered to co-assemble SN38-Arg with HPV16 E7 peptide (denoted as SFE), leveraging folate receptor overexpression to enhance tumor targeting. For CRC, analogous nanomicelles (SFA) were developed by substituting E7 with the MC38 neoantigen Adpgk peptide. Both nanomicelles demonstrated robust stability, efficient cellular uptake, and dual mechanisms of tumor control: DNA damage through Topoisomerase I inhibition and immune activation through dendritic cell maturation and cytotoxic T lymphocyte infiltration. Murine models of HPV-positive TC-1 and Adpgk-positive MC38 tumors validated the therapeutic potential of these formulations. Crucially, in a clinically relevant neoadjuvant setting, preoperative administration of SFE or SFA not only significantly reduced primary tumor burden but also prevented post-surgical recurrence and induced durable antitumor immune memory, without evident systemic toxicity. These findings establish a broadly applicable, “off-the-shelf” nanomicellar platform that synergistically couples SN38-based chemotherapy with personalized peptide vaccination, offering a versatile approach to improving surgical outcomes and long-term disease control across distinct tumor types.

## 2 Materials and methods

### 2.1 Materials

DSPE-PEG_2000_ (S25991, Shanghai Yuan Ye), DSPE-PEG_2000_-FA purchased from Pengshuo Biotechnology, SN38 (BD8481, Bide Pharmaceutical), Arginine (BD8577, Bide Pharmaceutical), HPV16 E7_44–62_ (44QAEPDRAHYNIVTFCCKCD62) purchased from GL Biochem. Adpgk (HLELASMTNMELMSSIVHQ) was purchased from Guoping Pharmaceutical Co., Ltd. Cell Counting Kit-8 (CCK8 kit) purchased from Glpbio (GK10001, USA). Unless otherwise specified, all chemicals were obtained from commercial sources and used without further purification. DMEM medium, RPMI 1640 medium, Penicillin/Streptomycin (P/S), 0.25% Trypsin-EDTA, Phosphate-Buffered Saline (PBS), and Fetal Bovine Serum (FBS) were purchased from Gibco Bio (USA).

### 2.2 Cells and animals

All cells were lines including TC-1, TC-1-LUC and DC2.4 purchased from the American Type Culture Collection (ATCC, USA). C57BL/6 mice (female, 4–6 weeks old) were obtained from the Guangdong Province Experimental Animal Center (Guangzhou, China), and all mice were housed in specific-pathogen-free (SPF) animal facilities. All animal experiments were conducted in accordance with the animal research guidelines provided by Guangzhou Medical University IACUC (Approval No. GY-2024–678).

### 2.3 Methods

#### 2.3.1 Synthesis of SN38-Arg

The synthetic route of SN38-Arg is shown in Fig. 2.

##### 2.3.1.1 Synthesis of Boc-SN38

SN38 (7-ethyl-10-hydroxycamptothecin) was weighed and dissolved in anhydrous dichloromethane in a 250 mL round-bottom flask. A small amount of anhydrous pyridine was added as a catalyst, and the reaction was stirred under nitrogen protection for 16 h at room temperature. After the reaction, 0.1 mol/L hydrochloric acid was used to extract the mixture, and the organic layer was retained. The extraction was repeated three times, followed by washing with saturated sodium bicarbonate solution to retain the organic layer. Anhydrous sodium sulfate was added for drying, the mixture was filtered, and the solvent was evaporated under reduced pressure. The resulting solid product, Boc-SN38, was stored at −20 °C.

##### 2.3.1.2 Synthesis of Boc-SN38-Arg

SN38 and arginine (Boc-Arg-OH, molar ratio 1:1.5) were dissolved in anhydrous dichloromethane in a 250 mL round-bottom flask. Catalysts including 4-dimethylaminopyridine (DMAP) and 1-ethyl-3-(3-dimethylaminopropyl) carbodiimide hydrochloride (EDCI) were added, and the reaction was stirred for 24 h. After the reaction, the mixture was concentrated under reduced pressure, yielding an oily product, which was stored at −20 °C.

##### 2.3.1.3 Deprotection to obtain SN38-Arg

Boc-SN38-Arg was dissolved in trifluoroacetic acid (TFA) in a 250 mL round-bottom flask and stirred for 3 h at room temperature. After the reaction, the solvent was evaporated under reduced pressure, and the resulting liquid was purified using a silica gel chromatography column (dichloromethane and ethyl acetate gradient elution). The purified light-yellow liquid was evaporated to dryness to obtain a light-yellow solid, which was then dissolved in ultrapure water and processed through multiple centrifugation steps to remove impurities. HPLC separation was carried out for purification using a binary gradient system. Mobile phase A was an aqueous solution of 0.05% formic acid, and mobile phase B was an acetonitrile solution of 0.05% formic acid. The flow rate was set at 1.0 mL/min with the following gradient program: the proportion of phase B was linearly increased to 95% over 11.00 min, maintained at 95% for 3.00 min (from 11.00 to 14.00 min), then rapidly reduced to the initial 5% within 0.50 min (at 14.50 min), and finally held at 5% for 0.50 min to re-equilibrate the column, giving a total run time of 15.00 min. The effluent was monitored at a wavelength of 254 nm by a UV detector. The resulting SN38-Arg solid was achieved by lyophilization and stored at −20 °C.

#### 2.3.2 Preparation of nanomicelle

E7 peptide, DSPE-PEG_2000_-FA, and DSPE-PEG_2000_ were mixed in a specific molar ratio (molar ratio of E7 peptide: DSPE-PEG_2000_-FA: DSPE-PEG_2000_ = 13:3:3) and dissolved in the ultrapure water, while SN38-Arg was dissolved in ethanol. The aqueous phase was added dropwise (~10 drops/min) to the organic phase under constant stirring for 15 min to form SFE nanomicelle. For the preparation of SFA nanomicelle, the same method was applied by loading the Adpgk peptide instead of the E7.

#### 2.3.3 Characterization of SFE nanomicelle

The morphology and size of the nanomicelle were observed using a JEM-1400PLUS transmission electron microscope (TEM). Dynamic light scattering (DLS) (Nano-ZS90) was used to measure the particle size distribution and ensure it met the expected range. The particle size and polydispersity index (PDI) were monitored over 7 days to assess stability. Unencapsulated drug molecules were separated by ultrafiltration, and the encapsulated drug content within the nanomicelle was quantified using the BCA assay kit (20201ES76, Yeasen Bio). The encapsulation efficiency and drug loading were calculated using the following formulas:

$\mathrm{Encapsulation Efficiency}=\left(\text{Mass of peptide in micelle}/\text{Total mass of peptide added}\right)\times 100\%.$

#### 2.3.4 Cellular uptake of SFE nanomicelle

Free E7-FAM (0.7 μg) and FAM-labeled SFE nanomicelle were added to DC2.4 cells. The uptake was observed at three timepoints: 0.5 h, 6 h, and 12 h. First, free drug was removed by washing with PBS. The nuclei of the cells were stained with DAPI (C1341M, Beyotime) for 15 min, followed by PBS washing. Cellular uptake was then analyzed using a Leica confocal microscope (Leica DLS). The distribution and intensity of the green fluorescence (representing internalized FAM-E7) were recorded to evaluate the uptake of SFE nanomicelle in DC2.4 cells.

DC2.4 were seeded in 6-well plates. After incubation with free E7-FAM and FAM-labeled SFE nanomicelle for 6 h, flow cytometry was performed to detect the uptake of different drugs by DC2.4.

#### 2.3.5 Primary mouse bone marrow-derived DCs (BMDCs) isolation

Mice were euthanized, and their hind limb bones were dissected. After removing the cartilage at both ends, the bone marrow was flushed using a pre-cooled 1640 culture medium. Bone marrow cells were collected by centrifugation and treated through multiple washes. These cells were resuspended in 10 mL culture medium. To promote the growth and differentiation of dendritic cells (DCs), cells were cultured in RPMI-1640 medium containing 10 μg/mL GM-CSF (Granulocyte-Macrophage Colony-Stimulating Factor) on day 3, and the medium was replenished with 10 mL of GM-CSF-containing RPMI-1640 medium. On day 6, cells were collected by centrifugation, resuspended in 10 mL GM-CSF RPMI-1640 medium, and further cultured for BMDC differentiation.

#### 2.3.6 Flow cytometry and ELISA analyzing BMDCs activation by SFE nanomicelle

BMDCs were isolated as the abovementioned procedures before seeding in the 6-well plates. After adding 1 μg/mL of free E7, SN38-Arg, and SFE nanomicelle for 24 h, the cells were digested with trypsin and washed with PBS. Cells were then blocked with 5% BSA for 15 min and stained with antibodies against surface markers of BMDCs, including CD11c (PCy5.5), CD80 (PE), CD86 (APC), MHC I (FITC) and MHC II (PE/Cyanine7). Flow cytometry was performed using the CytoFLEX Flow Cytometer (Beckman, USA), and data were analyzed with FlowJo software. The gating strategy is shown in Supplementary Fig. S1.

For cytokine levels, the supernatants of BMDCs were collected. ELISA kits were used to determine the concentrations of TNF-α, IL-1β, and IFN-γ. The assay involved forming antigen-antibody complexes using specific antibodies, followed by colour development with enzyme-linked antibodies, and measuring absorbance to determine cytokine concentrations.

#### 2.3.7 Western blot analysis in TC-1 cells

TC-1 cells were co-cultured with 1.0 μg/mL of free E7, SN38-Arg, or SFE nanomicelle. After 24 h, cells were lysed using RIPA buffer containing protease and phosphatase inhibitors (ST505, Beyotime). Protein concentrations were determined using a BCA Protein Assay Kit (20201ES, Yeasen Bio). The protein samples were separated by SDS-PAGE, then transferred to PVDF membranes (1620177, BioRad). Membranes were blocked and incubated overnight with primary antibodies for Top1 (38650S, CST) and Phospho-Histone H2A.X (5438S, CST). After incubation with secondary antibodies, protein bands were visualized using a gel imaging system and recorded.

#### 2.3.8 Apoptosis assay

TC-1 cells were co-cultured with 1 μg of free E7, SN38-Arg, or SFE nanomicelle. After 24 h, use trypsin to digest the adherent cells and collect them along with those suspended in the culture medium. Centrifuge the cell suspension, remove the supernatant, and then wash the cells with PBS before resuspending them. Next, add Annexin V-FITC and PI staining solutions, gently mix, and incubate in the dark at room temperature for a period, ranging from 10 to 15 min. Finally, inject the stained cell suspension into the flow cytometer for analysis.

#### 2.3.9 Biodistribution study of SFE nanomicelles in mice

Female BALB/c mice (6–8 weeks old) were randomized into Control, E7-FAM, and SFE groups (*n* = 3 per time point). Each mouse received a subcutaneous back injection of saline, E7-FAM, or SFE nanomicelles at 10 μg (fluorescent label). At 0.5, 6, 24, and 48 h post-injection, whole-body fluorescence images were taken using an in vivo imaging system. Mice were then euthanized, and major organs (heart, liver, spleen, lungs, kidneys) were harvested for ex vivo imaging to quantify tissue fluorescence signals.

#### 2.3.10 Treatment of subcutaneous tumor model with SFE nanomicelle

TC-1 cells were cultured to the logarithmic phase, and 1 × 10^6 cells were injected subcutaneously into mice. Once the tumor volume reached 80–120 mm^3^, the mice were randomly divided into five groups (*n* = 6): (1) PBS, (2) Free E7, (3) SN38-Arg, (4) SN38-Arg + E7, and (5) SFE. When the tumor volume reached approximately 100 mm^3^, the first dose was administered on day 7, followed by a second dose after a seven-day interval. During the experiment, the body weight and tumor volume of mice were monitored. On day 21, lymph nodes, spleen, and tumor tissue samples were collected for flow cytometry analysis.

#### 2.3.11 Therapeutic efficacy of SFA nanomicelles in MC38 CRC model

MC38 cells were cultured under optimal conditions to reach the logarithmic growth phase, ensuring adequate cell numbers and good viability. Subsequently, a suspension containing 1 × 10^6 MC38 cells was injected subcutaneously into the dorsal region of mice. When tumor volumes reached 80–120 mm^3^, the mice were randomly divided into five groups (*n* = 6): (1) PBS, (2) free Adpgk, (3) SN38-Arg, (4) SN38-Arg + Adpgk, and (5) SFA (micelles formed by SN38-Arg and lipid components loaded with Adpgk peptide). Subcutaneous administration was performed on days 9, 13and 17. During the experiment, mouse body weight and tumor volume were closely monitored. On day 21, lymph node, spleen and tumor tissue samples were collected for flow cytometry analysis.

#### 2.3.12 Treatment effect of SFE nanomicelle on recurrence and metastasis in the preoperative neoadjuvant therapy model

Mice were injected with 1 × 10^6 TC-1 cells subcutaneously, and once the tumor reached 50–80 mm^3^, they were randomly divided into five groups (*n* = 6). Treatments were administered on days 5, 7, and 9 via subcutaneous injections, followed by tumor resection. On the next day, TC-1 cells were re-inoculated into the contralateral side. Body weight and tumor volume were monitored, and on day 16 after surgery, lymph nodes, spleen, and tumor tissue samples were collected for flow cytometry analysis.

#### 2.3.13 Immunohistochemistry (IHC) and immunofluorescence (IF)

The tumor tissue sections were stained with hematoxylin and eosin (H&E) and TUNEL to detect histological changes. For IHC analyses on immune cell infiltration, the tissue sections were incubated with an anti-CD8 or anti-Foxp3 antibody (Servicebio). Finally, images were photographed using fluorescence microscopy.

#### 2.3.14 Flow cytometric analysis of tumor immune microenvironment (TIME)

Fresh tumors, lymph nodes, spleens were collected and subjected to anti-tumor immune response analysis using flow cytometry (CytoFLEX). In brief, samples were dissociated into single-cell suspensions and treated with red blood cell lysis buffer (R1010, Solabio) to remove red blood cells. Subsequently, the samples were blocked in 0.1% BSA (36101ES, Yeasen Bio.) in PBS and then incubated with relevant antibodies at room temperature for 1 h. For the characterization of T cells and DCs in tumors and spleens, staining was performed using (Table S1). Flow cytometry data were acquired using CytExpert software and processed using FlowJo software. Gating strategies are illustrated in Supplementary Fig. S2–4.

#### 2.3.15 Biochemistry assay of SFE in mice

Blood samples were collected from mice receiving various treatments, and plasma was separated by centrifugation. The samples were stored at low temperatures for stability before further blood biochemistry assays.

#### 2.3.16 Statistical analyses

All quantitative values are reported as the mean ± standard deviation (SD). Data were evaluated by one- or two-way analysis of variance (ANOVA) for comparison of multiple groups using GraphPad Prism 5 software. *p < 0.05, **p < 0.01, ***p < 0.001 and ****p < 0.0001 were considered as significant difference.

## 3 Results and discussion

### 3.1 Synthesis and characterization of SN38-Arg

The synthetic route is illustrated in Fig. 1. The successful preparation of the arginine-modified SN38, SN38-Arg was confirmed through nuclear magnetic resonance (NMR) spectroscopy (Fig.S5) and high-performance liquid chromatography (HPLC) (Fig. S6). SN38 itself is poorly soluble in both lipophilic and hydrophilic environments, by modifying it with Arg that conferred to an amphiphilic compound, which can significantly enhance the solubility and bioavailability for in vivo application.

**Fig. 1:**
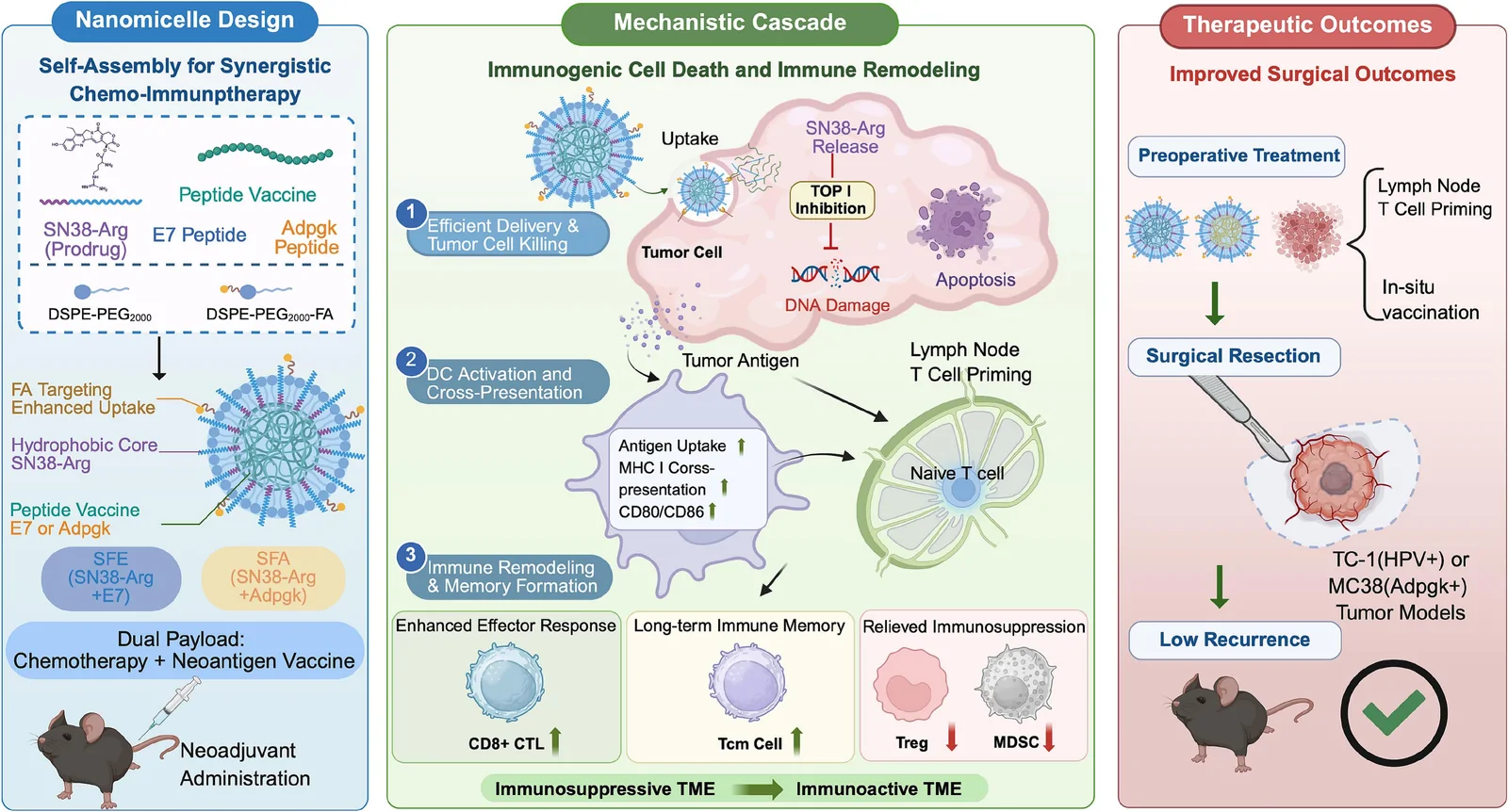
In this study, SN38 was modified with arginine to the amphiphilic SN38-Arg structure, co-assembled with neoantigen peptides E7 or Adpgk and DSPE-PEG_2000_-FA form the folate-targeted SN38-Arg nanomicelle named SFE or SFA, respectively. Its dual mechanism of action in immune activation and DNA damage was explored in depth in subcutaneous TC-1 and MC38 tumors, and the preoperative neoadjuvant chemoimmunotherapy schemes.

**Fig. 2:**
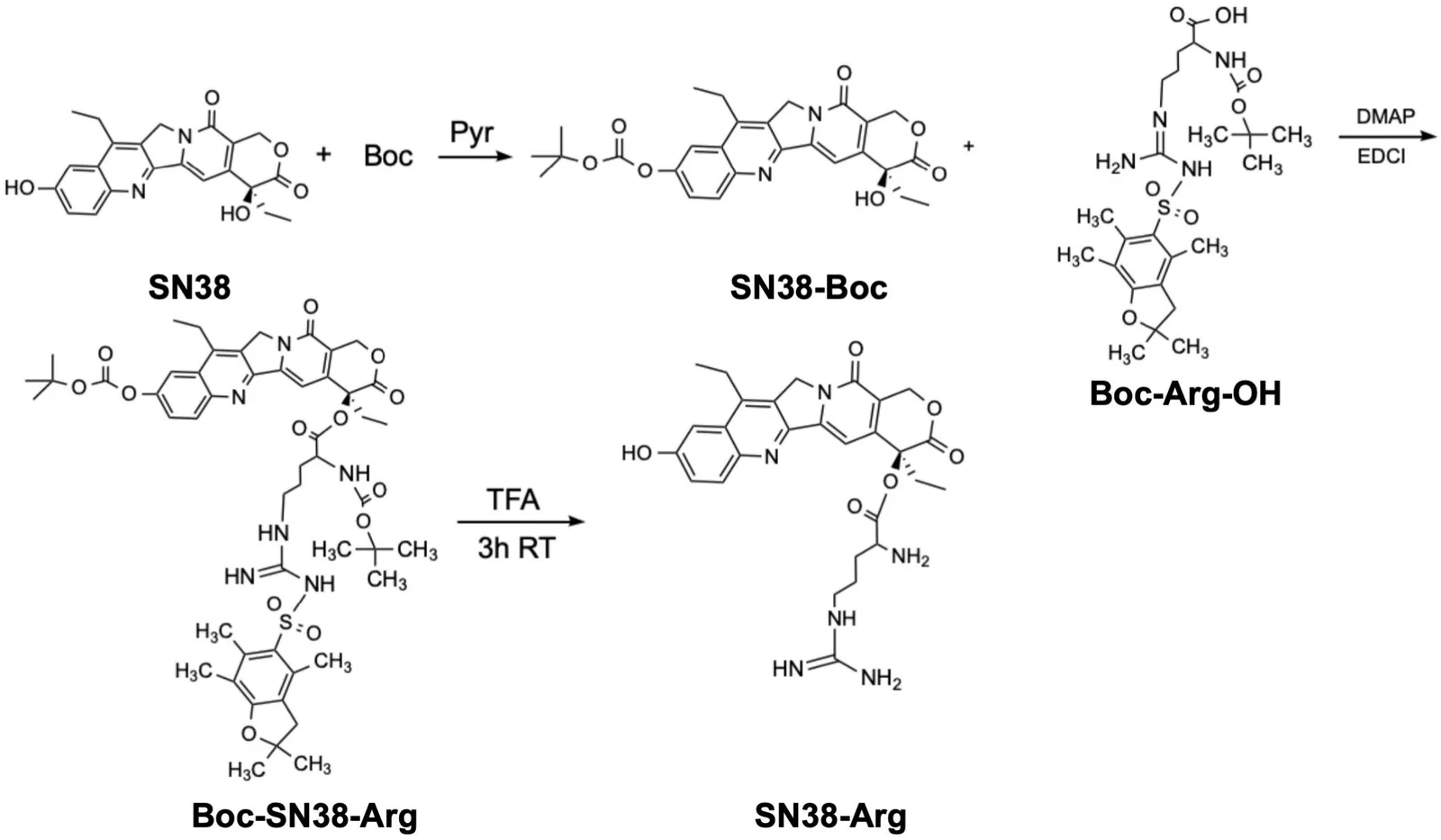
Synthetic route of SN38-Arg.

### 3.2 Synthesis and characterization of SFE nanomicelle

The SFE nanomicelle were successfully prepared by gradually adding the aqueous phase containing lipid components and the antigenic peptide E7 into the organic phase containing SN38-Arg. As shown in Fig. 3A, the SFE nanomicelle exhibited a uniform spherical morphology with a size of approximately 120 nm. Dynamic light scattering (DLS) analysis revealed that the average particle size of the SFE nanomicelle was 114.5 ± 0.53 nm, with a polydispersity index (PDI) of 0.373 ± 0.01 (Fig. 3B). The particle size and PDI remained stable with insignificant changes over a 7-day period (Fig. 3C). The encapsulation efficiency of E7 in the SFE nanomicelle was determined to be 63 ± 7% by BCA assay-determined standard curve (Fig. S7). These results indicate that stable nanomicelle with high drug loading efficiency were successfully obtained. Furthermore, the cumulative release of the peptide from the nanomicelles was monitored in PBS (pH 7.4) at 37 °C over 96 h. A sustained release profile was observed, with about 20% released within the first 12 h and about 70% released by 96 h (Fig. S8). These results indicate that the nanomicelles provide sustained peptide release over 96 h. Furthermore, the cytotoxicity of SN38-Arg and SFE nanomicelle against TC-1 cancer cells was evaluated by the CCK-8 assay (Fig. S9A). Furthermore, we examined the cytotoxicity and pro-apoptotic effect of SFE on DC2.4 cells. The IC50 value of SFE against DC2.4 cells was determined to be 23.99 μg/mL (Fig. S9B). At E7 and SN38-Arg concentrations of 1 μg/mL and 1.25 μg/mL, respectively, SFE did not markedly induce apoptosis in DC2.4 cells, showing only a 5% increase relative to the control group (Fig. S10).

**Fig. 3:**
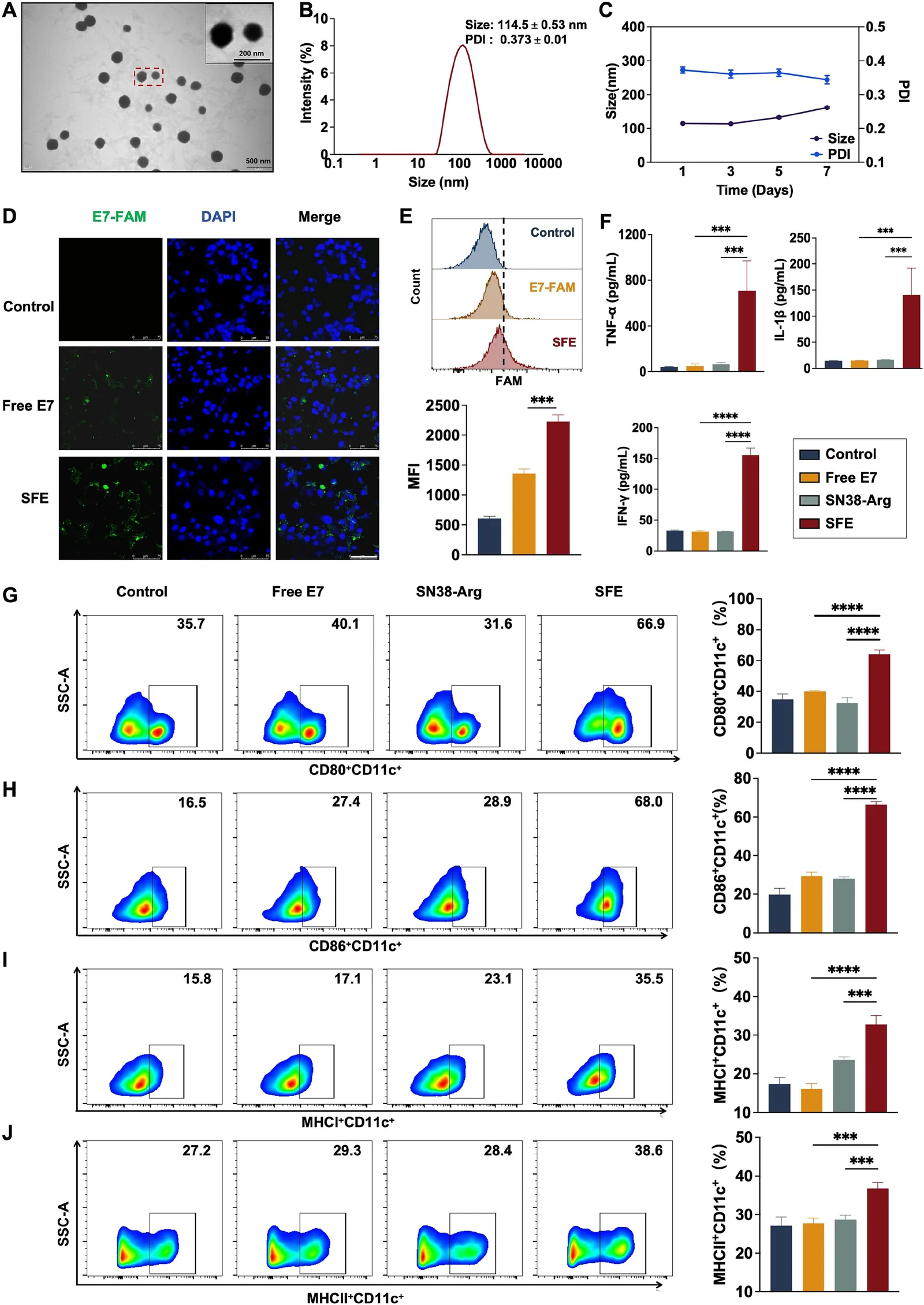
Characterization of SFE nanomicelle and its effect on the maturation of BMDC. (A) Representative TEM images of SFE nanomicelle. (B) DLS data of SFE nanomicelle. (C) Changes in the PDI and size of SFE nanomicelle over a 7-day period. (D) Representative confocal images showing cellular uptake after 6 h of treatment with Free E7 or SFE nanomicelle (scale bar: 75 μm). (E) Flow cytometry analysis of cellular uptake and quantitative data. (F) Secretion levels of cytokines TNF-α, IL-1β, and IFN-γ. (G) Flow cytometry analysis and statistical graph of surface marker CD80 expression. (H) Flow cytometry analysis and statistical graph of surface marker CD86 expression. (I) Flow cytometry plots and statistical analysis of surface marker MHC I expression. (J) Flow cytometry plots and statistical analysis of surface marker MHC II expression. *p < 0.05, **p < 0.01, ***p < 0.001, and ****p < 0.0001 represent significant differences.

The FAM labeled E7 peptide was used to track the cellular uptake profile in DC2.4 cells, and free E7-FAM and FAM-E7-labeled SFE nanomicelle were added to DC2.4 cells at different time points of 0.5 h, 6 h and 12 h (Fig. 3D, Fig. S11–12). The results demonstrated that the formation of nanomicelle significantly enhanced cellular uptake, and as time progressed, the uptake of SFE nanomicelle by cells increased. Flow cytometry quantitative analysis of MFI (Fig. 3E) further confirmed that SFE nanomicelle promoted cellular uptake of the antigenic peptide E7.

To investigate whether SFE nanomicelle can stimulate the activation and maturation of BMDCs, BMDCs were treated with PBS, Free E7 at 1 μg/mL, SN38-Arg at 1.25 μg/mL or SFE nanomicelle for 24 h. Flow cytometry analysis was performed to assess the expression of surface markers CD80 and CD86, which are indicative of BMDC maturation. The results showed that SFE nanomicelle significantly promoted the maturation of BMDCs (Fig. 3G-J). Additionally, ELISA was used to measure cytokine levels in the supernatant of BMDCs. The findings indicated that SFE nanomicelle enhanced the secretion of TNF-α, IL-1β, and IFN-γ, suggesting that SFE nanomicelle significantly boost the maturation capability of BMDCs (Fig. 3F).

### 3.3 Antitumor mechanism of SFE nanomicelle

Cell apoptosis was analyzed using an apoptosis detection kit and flow cytometry. The results showed that the apoptosis rate of TC-1 cells treated with SFE nanomicelle was significantly higher than that of the other groups, indicating that SFE have a stronger ability to inhibit tumor cell growth (Fig. 4A). Previous studies have demonstrated that SN38, a TOP1 inhibitor, suppresses tumor cell viability by inhibiting the expression of TOP1 enzyme protein that participating in the DNA uncoiling during the DNA replication, which may induce large amount of DNA mispairing and damage. Western blot analysis showed that SFE significantly reduced the expression of TOP1 protein (Fig. 4B), the increased expression of γ-H2AX, a DNA damage marker protein, was also confirmed by cellular immunofluorescence assay (Fig. 4C). These findings suggest that SFE nanomicelle can induce DNA damage in tumor cells.

**Fig. 4:**
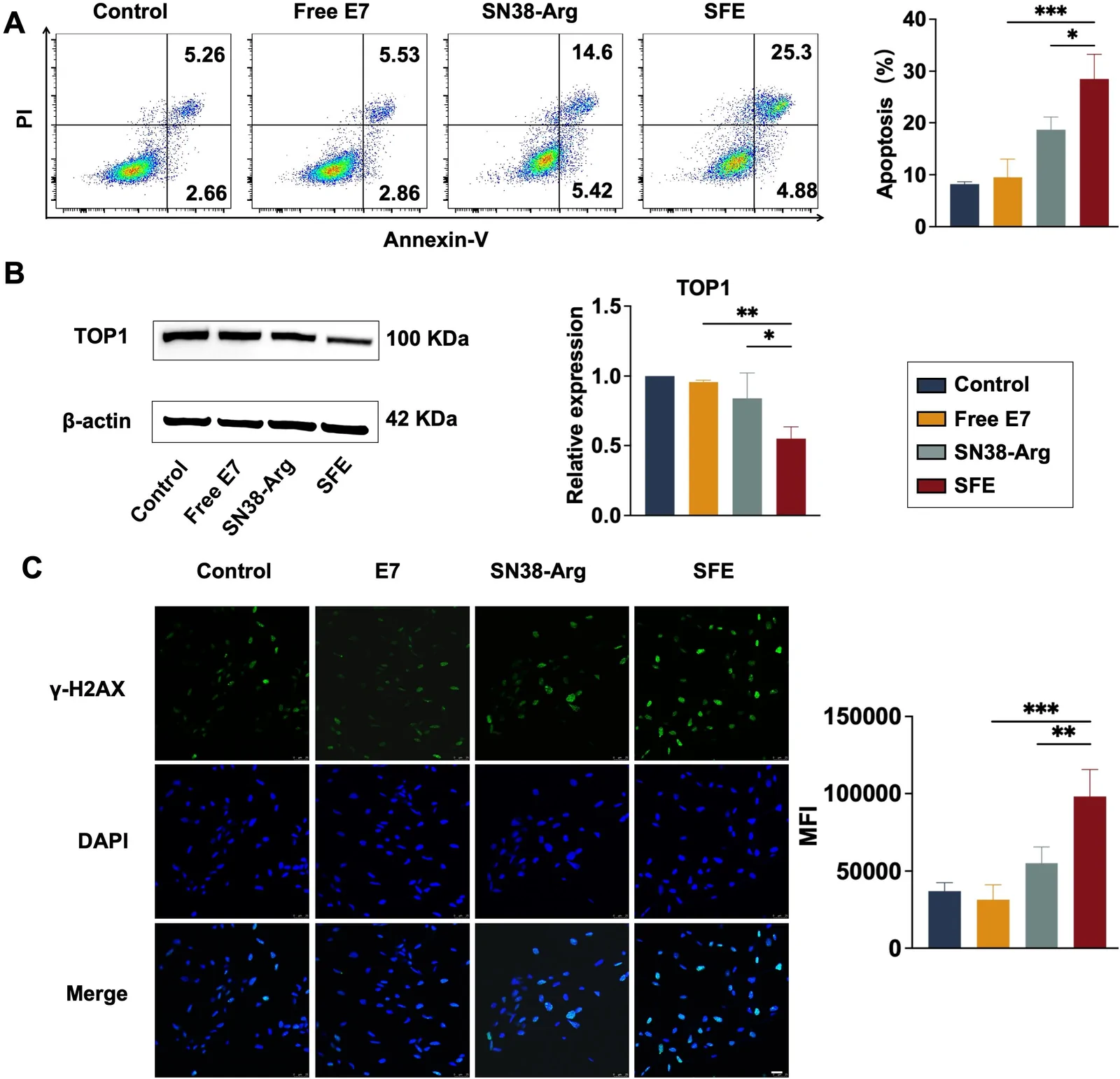
SFE nanomicelle induced apoptosis and DNA damage in TC-1 cells. (A) Representative images and quantitative analysis of cell apoptosis in TC-1 cells after 24-h treatment. (B) Western blot analysis of TOP1 and statistical quantification of protein expression. (C) Representative images and quantitative chart of γ-H2AX confocal immunofluorescence (scale bar: 25 μm). *p < 0.05, **p < 0.01, and ***p < 0.001 represent significant differences.

To further verify the biodistribution of SFE nanomicelle in vivo, the IVIS was carried out, as shown in the Fig. S13, E7-FAM fluorescence form SFE nanomicelle was observed at the lymph node with the strongest fluorescence intensity and longest retaining time within 6 h after drug administration, compared to other groups, indicating that SFE nanomicelle formulation contributed to the enhanced tumor accumulation and retention.

### 3.4 Antitumor efficacy of SFE vaccination in TC-1 tumor model

The therapeutic vaccination efficacy of SFE nanomicelle was evaluated in a TC-1 subcutaneous tumor model. As shown in Fig. 5A, on day 7 after tumor inoculation, mice were randomly divided into five treatment groups: PBS, free E7, SN38-Arg, SN38-Arg + E7, and SFE. Treatments were administered on days 7 and 15. Fig. 5B demonstrates that, compared to other treatment groups, SFE vaccination significantly inhibited tumor growth. Tumor growth was assessed (Fig. 5C), and the results showed that tumor growth was inhibited to varying degrees by different formulations (Fig. 5D). Among them, SFE vaccination exhibited the strongest tumor growth inhibitory effect, significantly prolonging the survival time in mice (Fig. 5E). Additionally, tumor weight (Fig. 5F) and spleen weight (Fig. S14A) measurements further confirmed the potential of SFE nanomicelle as an effective tumor treatment (Fig. 5G).

**Fig. 5:**
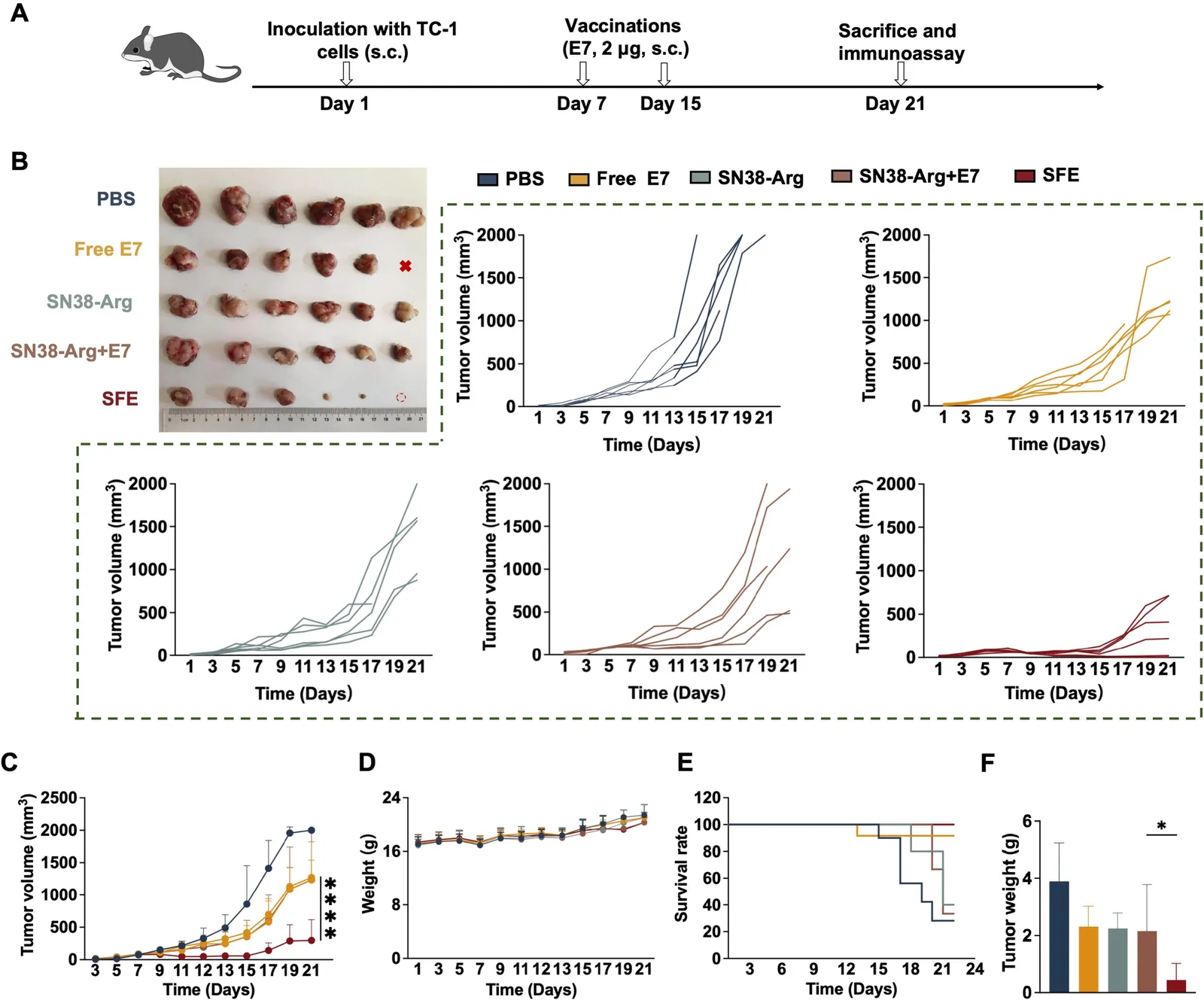
Antitumor efficacy of SFE immunization in the TC-1 subcutaneous tumor model. (A) Experimental workflow. (B) Tumor images of mice and the growth curves of mouse tumor in each group (x indicates mouse death, dashed circle indicates tumor eradication). (C) Tumor volume growth curves in mice. (D) The body wight of mice. (E) Mouse survival curve. (F) Tumor weight. *p < 0.05 and ****p < 0.0001 represent significant differences.

Subsequently, the antitumor mechanism was investigated using Western blotting to assess the expression levels of DNA repair-related proteins TOP1 and DNA damage marker γ-H2AX in the tumor tissue. The results (Fig. S14B) showed that SFE treatment effectively downregulated the expression of TOP1 while upregulating the expression of γ-H2AX. This led to the inhibition of DNA repair in tumor cells, promoting more accumulation of DNA damage and ultimately suppressing tumor cell growth.

The studies on BMDCs indicate that SFE nanomicelle could promote BMDCs maturation, enhance antigen presentation, and induce a series of immune responses, which were also successfully demonstrated in the in vivo antitumor experiment. To further verify the immune responses induced by SFE vaccination during tumor growth, lymphocytes from lymph nodes, spleen, and tumor tissues were collected and analyzed using flow cytometry. The results showed that, compared to the mixture group (SN38-Arg + E7), the proportion of CD8^+^ T cells and CTLs in the lymph nodes increased (Fig. 6A, Fig. S15A-B), while the proportion of immunosuppressive MDSCs and Treg cells decreased (Fig. S16A-B). Additionally, an increase of memory T cells proportion was observed (Fig. 6A, Fig. S16C-D), indicating that SFE plays a crucial role in amplifying antitumor immune responses and systemic immunity. To verify the antigen-specific T-cell response, we examined the frequency of E7 tetramer-positive T cells in lymph nodes. Flow cytometric analysis showed that the proportion of E7 tetramer^+^ CD8^+^ T cells in lymph nodes was higher in the SFE group than in the control group, indicating that SFE effectively induced E7-specific T cells in lymph nodes (Fig. S17). Similar immune responses were observed in splenic lymphocytes, with increased proportions of CD8^+^ T cells and memory T cells (Fig. 6B, Fig. S18A-D), and decreased proportions of MDSCs and Treg cells (Fig. S19A-B).

**Fig. 6:**
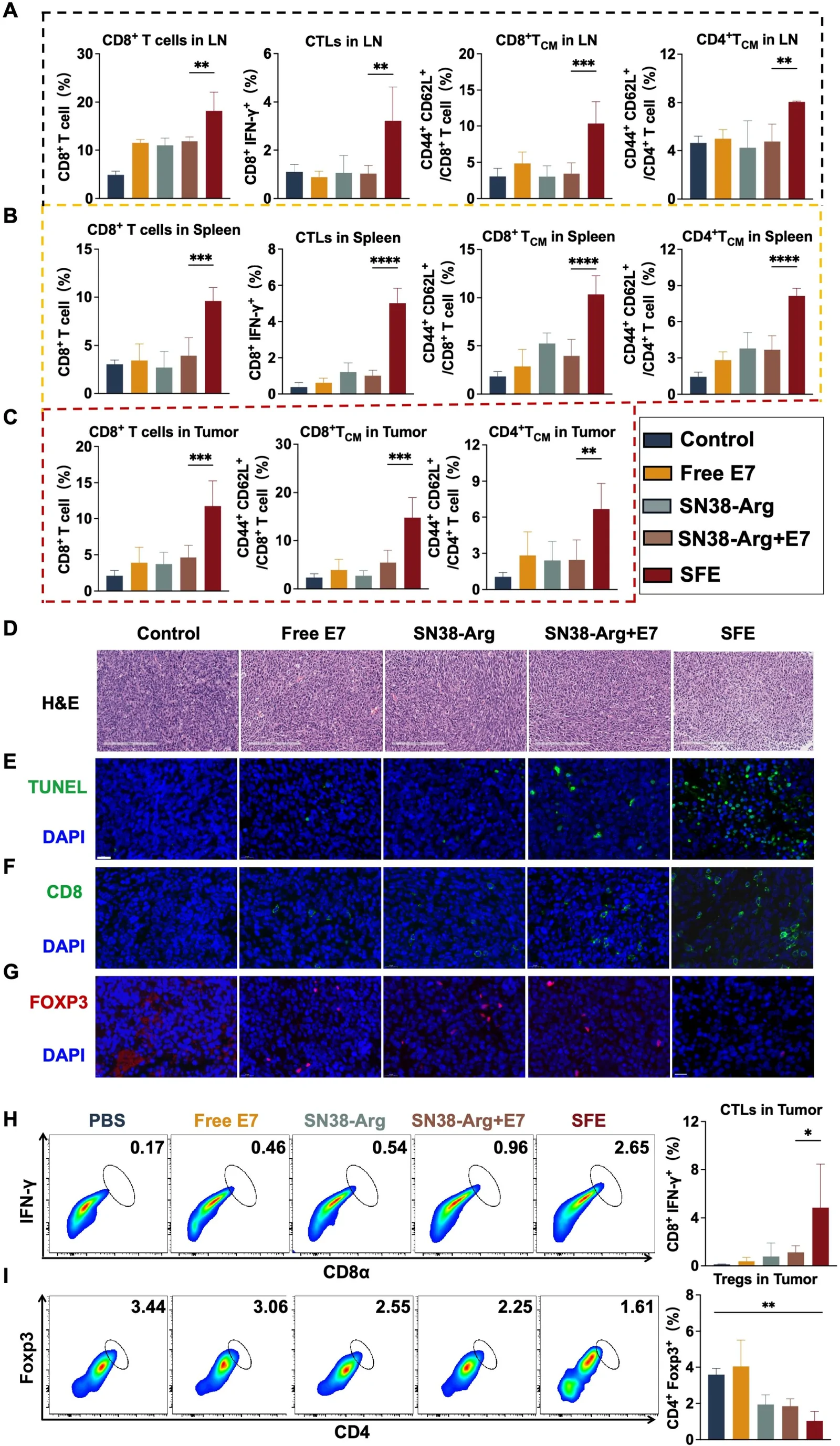
The immune responses induced by SFE vaccination in the TC-1 subcutaneous tumor model. (A) Quantification of CD8^+^ T cells, CD8^+^ IFN-γ^+^ cells, CD4^+^CD44^+^CD62L^+^ cells, and CD8^+^CD44^+^CD62L^+^ cells in the lymph nodes. (B) Quantification of CD8^+^ T cells, CD8^+^IFN-γ^+^ cells, CD4^+^CD44^+^CD62L^+^ cells, and CD8^+^CD44^+^CD62L^+^ cells in the spleen. (C) Quantification of CD8^+^ T cells, CD8^+^ IFN-γ^+^ cells, CD4^+^CD44^+^CD62L^+^ cells, and CD8^+^CD44^+^CD62L^+^ cells within the tumor. (D) Histological H&E staining image of tumor tissue (scale bar: 200 μm). (E) TUNEL (green) staining image showing tumor cell apoptosis (scale bar: 20 μm). (F) Representative immunohistochemical image showing CD8^+^ cell (green) infiltration in the tumor tissue (scale bar: 20 μm). (G) Representative immunohistochemical image showing Foxp3^+^ cell (green) infiltration in the tumor tissue (scale bar: 20 μm). Representative flow cytometry plots and the quantification of intratumoral CD8^+^IFN-γ^+^ CTLs (H) and CD4^+^Foxp3^+^ Treg cells(I). *p < 0.05, **p < 0.01, ***p < 0.001, and ****p < 0.0001 represent significant differences. (For interpretation of the references to colour in this figure legend, the reader is referred to the web version of this article.)

Subsequent analysis of tumor-infiltrating lymphocytes revealed that the similar reshaping effect of tumor immune microenvironment, compared to the mixture group (SN38-Arg + E7), SFE vaccination significantly increased lymphocyte infiltration into the tumor (Fig. 6C, Fig. S20A-D) and altered the immunosuppressive tumor microenvironment (Fig. S21A-B).

To further evaluate tumor damage across different treatment groups, tumor sections were subjected to H&E staining and immunofluorescence analysis. As shown in Fig. 6D, the SN38-Arg + E7 group exhibited densely packed cells with normal nuclear morphology, whereas the SFE group displayed sparse cell distribution and nuclear shrinkage, suggesting the highest level of proliferation inhibition in the SFE group. This finding was corroborated by TUNEL assay results (Fig. 6E), which showed the highest tumor cell apoptosis in the SFE group.

Additionally, immunofluorescence analysis revealed that SFE treatment elevated the proportion of CD8^+^ T cells and reduced Treg cells (Fig. 6F-G). These results indicate that SFE nanomicelle can remodel the tumor immune microenvironment, promoting immune cell infiltration into the tumor. This finding is consistent with flow cytometry data showing changes in CD8^+^ T and Treg cell populations within the tumor (Fig. 6H-J).

Finally, to assess the safety profile of SFE, H&E staining of the major organs (heart, liver, spleen, lung, and kidney), and blood biochemical assay were performed (Fig. S22A). The biochemical parameters of blood samples from the different treatment groups were also analyzed, and no significant differences were observed between SFE-treated and PBS-treated mice (Fig. S22B). Furthermore, no significant changes were detected in the major tissues and organs of SFE-treated mice compared to PBS-treated controls. These results provide further evidence that SFE possesses sufficient biosafety.

### 3.5 The therapeutic effect of SFA vaccination in Adpgk-MC38 CRC model

Above studies have confirmed that SN38-Arg nanomicelles loaded with the E7 antigen (SFE) exhibit significant efficacy in a cervical cancer model. To validate the versatility of this platform technology, the CRC-specific neoantigen Adpgk was selected as the model antigen loading in the carrier forming another nanomicelle formulation, SFA. This study further demonstrated the therapeutic effects of SFA nanomicelles using the Adpgk-MC38 model. To this end, the SFA vaccinations were applied as the scheme shown in Fig. 7A when tumor volumes reached 80–100 mm^3^. The tumor growth (Fig. 7B-C) and mouse bodyweight (Fig. S23A) were monitored every two days. Compared to other groups, the SFA group exhibited significant tumor growth inhibition and improved the survival rate of mice while keeping the body weight stable (Fig. S23B). Tumor weight measurements (Fig. 7D) further confirmed that SFA vaccination effectively suppressed tumor progression.

**Fig. 7:**
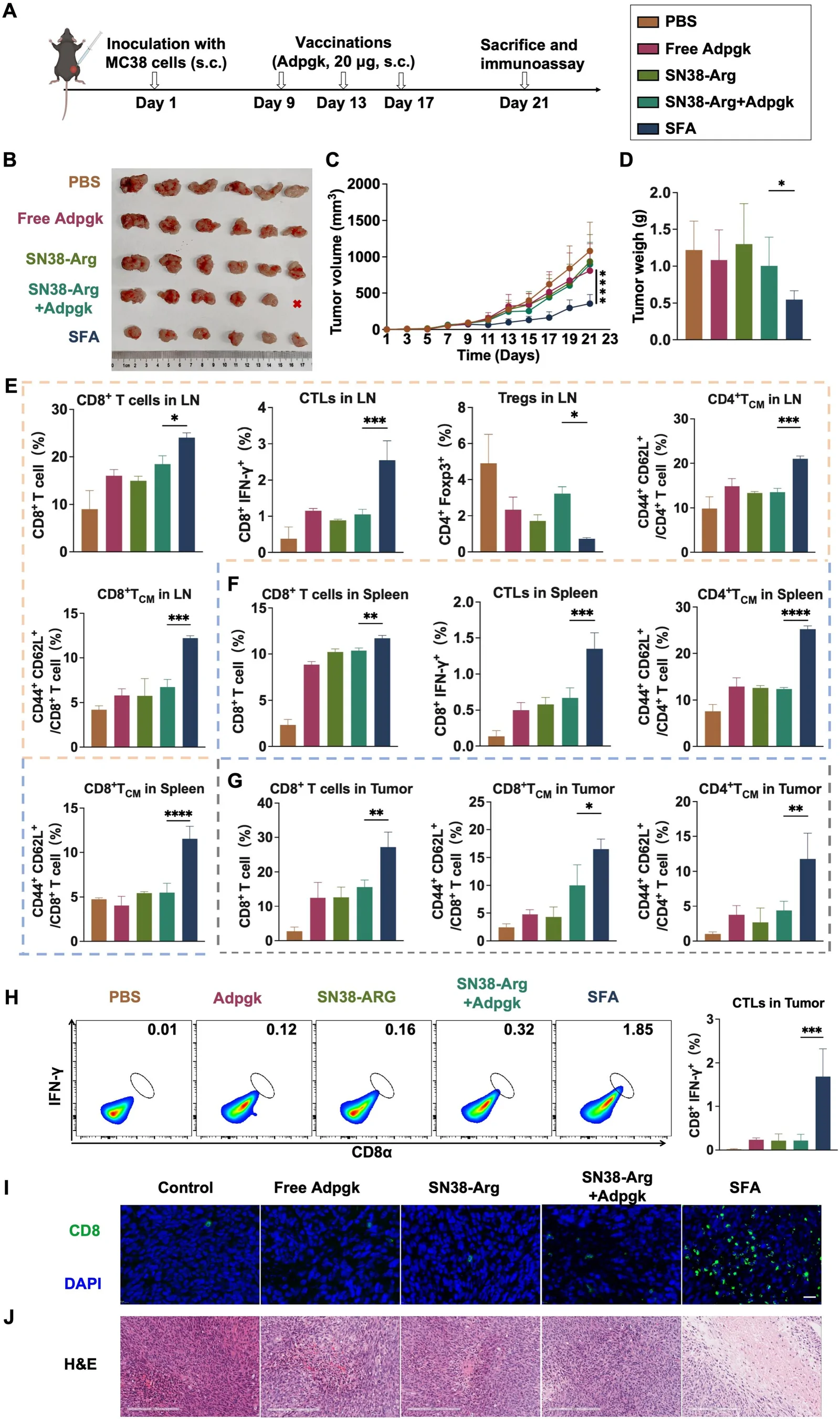
Therapeutic effect of SFA vaccination on subcutaneous MC38 CRC model. (A) Experimental flow chart. (B) Photographs of tumors. (x indicates mouse death) (C) Tumor volume growth curve. (D) Bodyweight curve. (E) Analysis of the immune response in lymph nodes of SFA vaccinated mice. (F) Immunological response analysis of SFA vaccination in the spleen. (G) Immunological response analysis in tumors of SFA vaccinated mice. (H) Representative flow cytometry plots and the corresponding quantification of intratumoral CD8^+^IFN-γ^+^ CTL cells. (I) Representative immunohistochemical image showing tumoral infiltration of CD8^+^ T cells (green) (scale bar: 20 μm). (J) Histological H&E staining image of tumor tissue (scale bar: 200 μm). *p < 0.05, **p < 0.01, ***p < 0.001, and ****p < 0.0001 represent significant differences. (For interpretation of the references to colour in this figure legend, the reader is referred to the web version of this article.)

Subsequently, flow cytometry was employed to analyze immune cell populations in lymph nodes (Fig. 7E). The results demonstrated that, compared to the PBS group, the SFA group showed an increased proportion of CD8^+^ T lymphocytes and cytotoxic T lymphocytes (CTLs) (Fig. S24A-B) in lymph nodes, while the proportions of regulatory T cells (Tregs) decreased (Fig. S24C) and MDSCs (Fig. S25) decreased as well. Additionally, elevated levels of memory T cells were observed (Fig. S26A-B), suggesting that SFA plays a crucial role in immune activation, amplifying systemic antitumor immune responses. A similar immunomodulatory effect was observed in splenic lymphocytes (Fig. 7F), with increased CD8^+^ T cells (Fig. S27A-B) and memory T cell populations (Fig. S27C-D), alongside reduced the proportions of immunosuppressive MDSCs and Tregs (Fig. S28). Further analysis of tumor-infiltrating lymphocytes revealed that, compared to the PBS group, SFA enhanced lymphocyte infiltration of CD8^+^ T cells and even memory T cells into tumors (Fig. 7G-I) and remodeled the immunosuppressive tumor microenvironment (Fig. S29B-C). Notably, the proportion of tumoral cytotoxic T cells increased significantly, which is important in immune killing of tumor cells, (Fig. 7H and Fig. S31A) while the proportion of immunosuppressive cells decreased (Fig. S30).

To evaluate tumor damage across different treatment groups, tumor sections were subjected to H&E staining and immunofluorescence analysis. As shown in Fig. 7J, the PBS group exhibited densely packed cells with normal nuclear morphology, whereas the SFA group displayed sparse cell distribution and nuclear shrinkage, suggesting the highest level of proliferation inhibition in the SFE group. This finding was corroborated by TUNEL assay results (Fig. S29A), which showed the highest tumor cell apoptosis in the SFE vaccinated group. Moreover, the proportion of memory T cells within tumors also increased (Fig. S31B-C). These data demonstrated that SFA vaccination-based immunotherapy shares the similar immunomodulation effects as the SFE, which further highlights this amphiphilic argininated SN38 based nanomicelle could be applied universally to treat other tumor model in this chemo-synergized vaccination.

### 3.6 Study on SFE neoadjuvant chemo-immunotherapy for minimizing post-surgery metastasis and recurrence

In the subcutaneous tumor model, the accumulation of CD8^+^ CTLs in the tumor was increased, and SFE vaccination generated post-treatment may amplify systemic immune responses, thereby eradicating metastatic tumor cells after surgery. To further validate this hypothesis, a model of tumor metastasis and recurrence after surgical resection was constructed (Fig. 8A), in which three SFE vaccinations were applied before surgical removal of the first tumor two days after the last vaccination, and live TC-1 cells were inoculated on the contralateral side the next day to simulate the postsurgical metastasis. In this model, the presurgical treatment with SFE vaccination-based neoadjuvant chemo-immunotherapy was demonstrated to significantly inhibit tumor metastasis and recurrence after surgery (Fig. 8B-D, Fig. S32A), leading to total tumor suppression in two out of six mice. Additionally, this effect was confirmed by tumor weight and spleen weight (Fig. S32B-C).

**Fig. 8:**
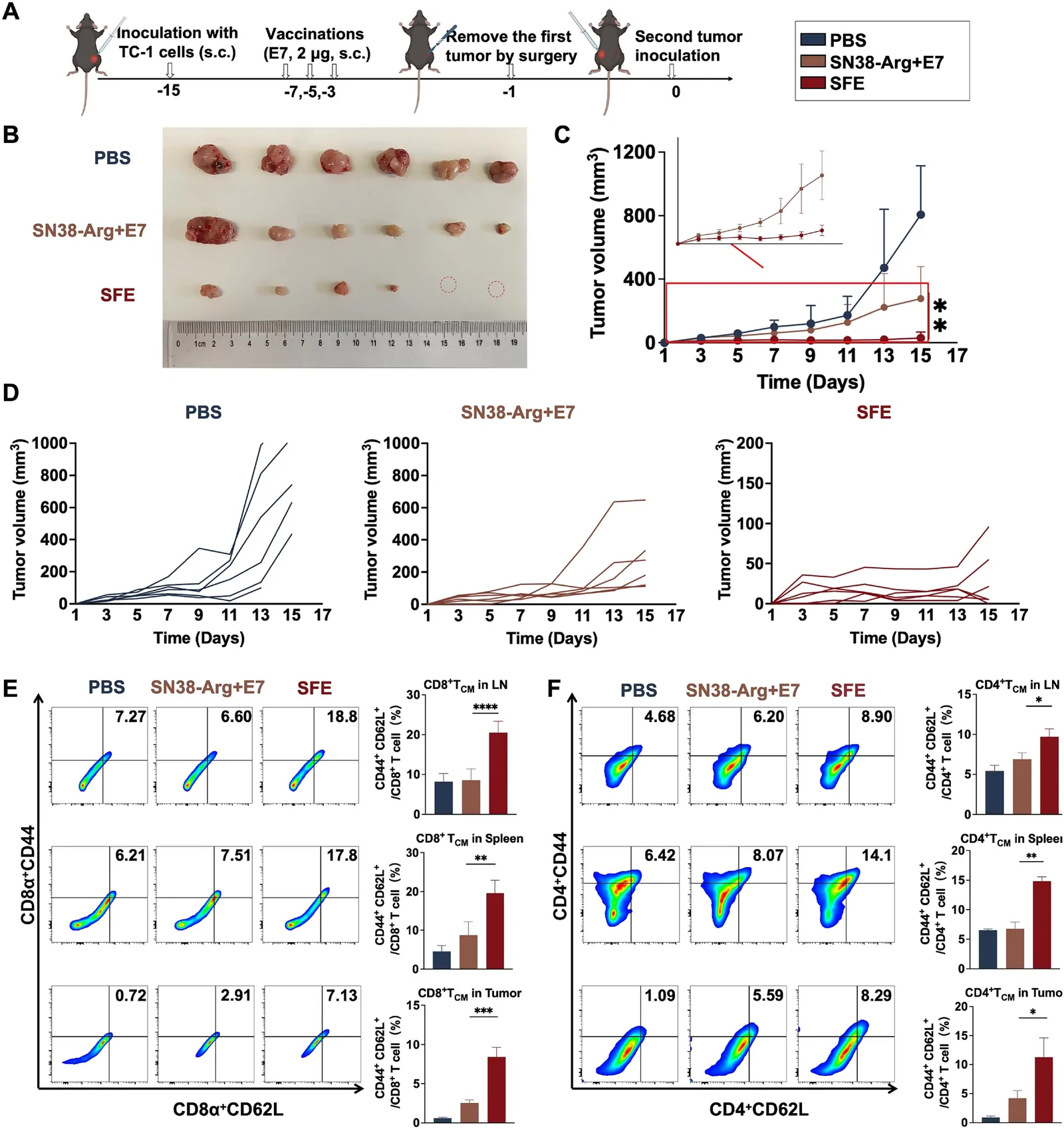
The study of inhibitory effect of SFE neoadjuvant vaccination on the postsurgical tumor metastasis and recurrence. (A) Experimental flowchart. (B) The images of secondary tumors (the dashed circle indicates no tumor growth). (C) Tumor growth curves. (D) Tumor growth curves of each group. Representative flow cytometry plots and quantitative analysis of the central memory T cells: (E) CD8^+^T_CM_ cells and (F) CD4^+^T_CM_ cells in lymph nodes, spleen, and tumors, respectively.

Lymphocytes in the spleen, lymph nodes, and tumor tissues were further analyzed to investigate whether SFE treatment could induce immune memory protection. As shown in Fig. 8E-F, in mice that underwent surgery followed by SFE treatment, the proportions of memory T cells in the lymph nodes and spleen were significantly higher compared to the PBS-treated surgery group. These results indicate that subcutaneous vaccination using SFE enhances immune memory responses in mice and effectively suppresses post-surgical tumor metastasis and recurrence.

## 4 Discussion

In an existing phase III clinical trial (Wang et al., 2019b), irinotecan, a prodrug of SN38, was combined with capecitabine and concurrent radiotherapy as preoperative chemoradiotherapy for patients with advanced rectal cancer. Surgical resection was performed eight weeks after treatment completion, thereby improving patient survival. In addition, in a phase II clinical trial (Rogers et al., n.d.), nanoliposomal irinotecan combined with 5-fluorouracil and oxaliplatin was used to treat pancreatic ductal adenocarcinoma, and the incidence of major complications within 30 days after resection was evaluated. These results suggest that the use of SN38 prodrugs as preoperative neoadjuvant therapy is a promising therapeutic strategy with potential for clinical translation.

Despite these encouraging results, we acknowledge several limitations that merit future investigation. Long-term safety and immunogenicity studies are imperative, extended toxicological and biodistribution assessments over at least 6 months (Tong et al., 2024; Torchelsen et al., 2026), alongside evaluation of anti-carrier antibody responses, should be conducted to fully establish the translational feasibility of this system. Additionally (Li et al., 2021; Yu et al., 2026), the underlying mechanisms governing the observed antitumor immunity remain to be elucidated, and future work using transcriptomic and proteomic profiling of the tumor microenvironment and peripheral immune cells will be pursued to dissect the complex regulatory networks involved. Future studies including transcriptomic analysis of tumor infiltrating lymphocytes and arginase activity assays are warranted to elucidate this mechanism. Collectively, this synergized chemo-vaccination approach demonstrates broad application prospects and considerable potential in both chemo-immunotherapy and preoperative neoadjuvant therapy for distinct tumor types (Fallarino and Blank, 2026; Khleif and Gupta, 2025). Future work will focus on further optimizing the treatment regimen and clarifying the mechanistic basis, aiming to provide more precise and effective therapeutic options for cancer patients.

## 5 Conclusion

This study successfully developed an innovative nanomecille delivery system, namely the lipid -based SN38-Arg nanoparticle loaded with neoantigen peptides and achieved remarkable results in the chemo-immunotherapy and preoperative neoadjuvant therapy of immuno-cold tumors. In terms of chemo-immunotherapy, SFE nanomicelle demonstrated robust anticancer activity. The SN38 drug component loaded in them effectively inhibited the proliferation and spread of tumor cells, while the delivery of peptide antigens activated the systematic and memory immune response of antitumor effect. This combined strategy of chemotherapy and immunotherapy not only significantly improved the therapeutic outcome of vaccination but also effectively reduced the toxicity and side effects of the drugs, providing a safer and more effective treatment strategy. By administering the nanomicelle vaccination before surgery, the tumors were successfully reduced and even totally suppressed, thereby increasing the surgical resection rate and radical resection rate, significantly decreasing the risk of tumor recurrence and metastasis, providing strong support for patients' postoperative rehabilitation and long-term survival. In summary, this synergized chemo-vaccination approach has shown broad application prospects and great potential in the chemo-immunotherapy and preoperative neoadjuvant therapy of distinct tumors. In the future, the mechanism of action will be explored and optimal treatment regimen of such nanomicelle, aiming to provide more precise and effective treatment options.

## CRediT authorship contribution statement

**Jiaxin Guo:** Formal analysis, Investigation, Methodology, Writing – original draft. **Miaoyan Liu:** Project administration, Methodology, Writing – original draft, Validation. **Ping Wan:** Funding acquisition, Resources, Formal analysis. **Yang Zhou:** Software, Investigation. **Dexu Luo:** Visualization, Methodology. **Xueheng Wu:** Visualization, Data curation. **Zhiyi Zhou:** Software, Methodology. **Xiaowei Huang:** Resources, Methodology. **Chengzhi Jin:** Writing – review & editing, Supervision, Project administration. **Xin Deng:** Methodology, Formal analysis, Investigation, Project administration. **Jialong Qi:** Supervision, Methodology, Funding acquisition, Writing – review & editing. **Wenjie Chen:** Writing – original draft, Funding acquisition, Writing – review & editing, Supervision.

## Ethics declaration

This study was conducted in accordance with the ARRIVE (Animal Research: Reporting of In Vivo Experiments) guidelines. This study was approved by the Guangzhou Medical University IACUC. (Approval No. Approval No. GY-2024–678).

## Funding

This work was supported by the 10.13039/501100001809National Natural Science Foundation of China (No.82400017, 82572623), the fund (2023A1515110028) from the Guangdong Province Science and Technology Bureau, Guangdong Basic and Applied Basic Research Foundation (No. 2023A1515110887) and the Open Project of the 10.13039/100013262State Key Laboratory of Respiratory Disease (SKLRD-OP-202409). This work was also supported by Yunnan Province Xingdian Talent Support Program for Young Professionals (No. KH-2025-XDYC-QNRC-01) and Yunnan Health Training Project of High Level Talents (No. H-2024064), and the The First People's Hospital of Yunnan Province Talent Project program (No. KHYJ-2025-04-02) and Yunnan Province Clinical Medical Center for Digestive System Diseases Open Project (No. 2024YNLCYXZX116/250). Dr. Chen thanks the Pearl River Rising Scholar Fellowship and thanks the Scientific Research Center of Guangzhou Medical University for providing experimental facilities and technical support.

## Declaration of competing interest

The authors declare that they have no known competing financial interests or personal relationships that could have appeared to influence the work reported in this paper.

## Data Availability

All data needed to evaluate the conclusions in the paper are present in the paper and/or the Supplementary Materials.
