# Supplementary material for: The lipid nanomicelles incorporating peptide vaccine and SN38 prodrug potentiate neoadjuvant chemo-immunotherapy
Source: Int J Pharm X. 2026 Oct 9;12:100681. doi: 10.1016/j.ijpx.2026.100681 (PMC13693331; doi:10.1016/j.ijpx.2026.100681)
Supplement: Supplementary file 1 [file mmc1.docx]

Supplementary information

**The Lipid Nanomicelles Incorporating Peptide Vaccine and SN38 Prodrug Potentiate Neoadjuvant Chemo-Immunotherapy**

**Figure S1.** Gating strategy for flow cytometry of mature BMDCs cells. (CD80^+^, CD86^+^, MHC I^+^ and MHC II^+^).

**Figure S2.** Gating strategy for flow cytometry of immune cells in mouse lymph nodes. (Central memory T cells, CD8^+^T cells, Cytotoxic T cells (CTL), Regulatory T cells (Tregs), Myeloid-derived suppressor cells (MDSCs))

**Figure S3.** Gating strategy for flow cytometry of splenic immune cells. (Central memory T cells, CD8^+^T cells, Cytotoxic T cells (CTL), Regulatory T cells (Tregs), Myeloid-derived suppressor cells (MDSCs))

**Figure S4.** Gating strategy for flow cytometry of intratumoral immune cells. (Central memory T cells, CD8^+^T cells, Cytotoxic T cells (CTL), Regulatory T cells (Tregs), Myeloid-derived suppressor cells (MDSCs), from top to bottom row)

**Figure S5.** Synthesis and Characterization of SN38-Boc and SN38-Arg.(A) Proton nuclear magnetic resonance (NMR) spectrum of SN38-Boc. (B) Mass spectrometry (MS) spectrum of SN38-Boc. (C) Proton nuclear magnetic resonance (NMR) spectrum of SN38-Arg. (D) Mass spectrometry (MS) spectrum of SN38-Arg.

SN38-Boc : ^1^H NMR (400 MHz, DMSO-*d*_6_) δ 8.20 (d, *J* = 9.2 Hz, 1H), 8.09 (s, 1H), 7.74 (dd, *J* = 9.2, 2.4 Hz, 1H), 7.33 (s, 1H), 6.54 (s, 1H), 5.44 (s, 2H), 5.33 (s, 2H), 3.20 (q, *J* = 7.3 Hz, 2H), 1.87 (hept, *J* = 7.0 Hz, 2H), 1.54 (s, 9H), 1.29 (t, *J* = 7.5 Hz, 3H), 0.88 (t, *J* = 7.3 Hz, 3H).

SN38-Arg :^1^H NMR (400 MHz, DMSO-*d*_6_) δ 8.39 (s, 1H), 8.18 (s, 1H), 8.08 – 7.97 (m, 1H), 7.46 – 7.36 (m, 4H), 7.15 – 6.98 (m, 1H), 5.45 (d, *J* = 27.4 Hz, 1H), 5.35 – 5.14 (m, 2H), δ 5.05 (d, *J* = 16.0 Hz, 1H), 4.85 (d, *J* = 15.8 Hz, 1H), 3.56 (dd, *J* = 7.6, 3.4 Hz, 1H), 3.50 (d, *J* = 7.1 Hz, 1H), 3.17 (s, 1H), 3.07 (d, *J* = 7.8 Hz, 4H), 2.40 – 2.01 (m, 2H), 2.00 – 1.69 (m, 2H), 1.68 – 1.48 (m, 2H), 1.30 (t, *J* = 7.6 Hz, 3H), 1.02 – 0.85 (m, 3H).

**Figure S6.** HPLC chromatogram of SN38-Arg.

**Figure S7.** Standard curve of E7.

**Figure S8.** In vitro release profile of SFE. The release behavior of SFE was investigated by the dialysis method at 37 °C in PBS (pH 7.4). At predetermined time points, samples were collected, and the concentration of E7 peptide in SFE was determined by BCA assay. The cumulative release was then calculated. Data are presented as mean ± SD (n = 3).

**Figure S9.** (A) Cytotoxicity of SN38-Arg and SFE nanomicelle against TC-1 cells. (B) Cytotoxicity of SN38-Arg and SFE nanomicelle against DC2.4 cells.

**Figure S10.** Representative images and quantitative analysis of cell apoptosis in DC2.4 cells after 24-hour treatment.

**Figure S11.** Cellular uptake of SFE after 0.5 h of incubation in DC2.4. (scale bar: 75 μm).

**Figure S12.** Cellular uptake of SFE after 0.5 h of incubation in DC2.4. (scale bar: 75 μm)

**Figure S13.** (A) Fluorescence intensity maps of various organs of mice at 0.5 h, 6 h, 24 h and 48 h after subcutaneous injection. (B) Quantitative graphs showing fluorescence intensity in various organs and tumors at different time points after drug administration.

**Figure S14.** Therapeutic effect of SFE nanomicelle in the TC-1 subcutaneous tumor model. (A) Spleen weight in mice. (B) Western blot analysis of DNA damage-related proteins in tumor cells.

**Figure S15.** Representative flow cytometry plots of immune cells in lymph nodes from the TC-1 tumor model. (A) CD8^+^ T cells. (B) CD8^+^IFN-γ^+^ cells. (C) CD4^+^CD44^+^CD62L^+^ cells. (D) CD8^+^CD44^+^CD62L^+^ cells.

**Figure S16.** SFE nanomicelle improve the immunosuppressive microenvironment in the TC-1 subcutaneous tumor model. (A) Quantification of CD4^+^Foxp3^+^ cells. (B) Quantification of CD11b^+^Gr-1^+^ cells in lymph nodes.

**Figure S17.** The analysis of E7-specific CD8^+^ T cells in the lymph nodes.

**Figure S18.** Representative flow cytometry plots of immune cells in the spleen from the TC-1 tumor model. (A) CD8^+^ T cells. (B) CD8^+^IFN-γ^+^ cells. (C) CD4^+^CD44^+^CD62L^+^ cells. (D) CD8^+^CD44^+^CD62L^+^ cells.

**Figure S19.** SFE nanomicelle improve the immunosuppressive microenvironment in the TC-1 subcutaneous tumor model. (A) Quantification of CD4^+^Foxp3^+^ cells in the spleen. (B) Quantification of CD11b^+^Gr-1^+^ cells in the spleen

**Figure S20.** Representative flow cytometry plots of immune cells in the tumor from the TC-1 subcutaneous tumor treatment model. (A) CD8^+^ T cells. (B) CD8^+^IFN-γ^+^ cells. (C) CD4^+^CD44^+^CD62L^+^ cells. (D) CD8^+^CD44^+^CD62L^+^ cells.

**Figure S21.** SFE nanomicelle improve the immunosuppressive microenvironment in the TC-1 subcutaneous tumor model. (A) Quantification of CD4^+^Foxp3^+^ cells in the tumor. (B) Quantification of CD11b^+^Gr-1^+^ cells in the tumor.

**Figure S22.** Biosafety studies of nanovaccines. (A) H&E staining of major organs (heart, liver, spleen, lung, kidney) of PBS, Free E7, SN38-Arg, SN38-Arg+E7, SFE treated mice (scale bar: 200 μm). (B) in vivo assessment of systemic toxicity after treatment. The changes of serum ALT, UR, AST, CR and CK were analyzed.

**Figure S23.** Therapeutic effect of SFA nanomicelle on MC38 subcutaneous tumor model. (A) Mouse body weight. (B) Mouse survival curve.

**Figure S24.** Representative flow cytometry plots of immune cells in lymph nodes from the MC38 subcutaneous tumor treatment model. (A) CD8^+^ T cells. (B) CD8^+^IFN-γ^+^ cells. (C) CD4^+^Foxp3^+^ cells.

**Figure S25.** Representative flow cytometry plots of MDSCs (CD11b^+^Gr-1^+^ cell) in lymph nodes from the MC38 subcutaneous tumor model.

**Figure S26.** Representative flow cytometry plots of immune cells in lymph nodes from the MC38 subcutaneous tumor treatment model. (A) CD4^+^CD44^+^CD62L^+^ cells. (B) CD8^+^CD44^+^CD62L^+^ cells.

**Figure S27.** Representative flow cytometry plots of immune cells in the spleen from the MC38 subcutaneous tumor treatment model. (A) CD8^+^ T cells. (B) CD8^+^IFN-γ^+^ cells. (C) CD4^+^CD44^+^CD62L^+^ cells. (D) CD8^+^CD44^+^CD62L^+^ cells.

**Figure S28.** Representative flow cytometry plots of immune cells in the spleen from the MC38 subcutaneous tumor treatment model. (A) Quantification of CD4^+^Foxp3^+^ cells in the spleen. (B) Quantification of CD11b^+^Gr-1^+^ cells in the spleen.

**Figure S29.** (A) TUNEL (green) staining image showing tumor cell apoptosis (scale bar: 20 μm). (B) Representative immunohistochemical image showing Foxp3^+^ cell (green) infiltration in the tumor tissue (scale bar: 20 μm). (C) Quantification of CD4^+^Foxp3^+^ cells in the tumor.

**Figure S30.** Representative flow cytometry plots of immune cells in the tumor from the MC38 subcutaneous tumor treatment model. Quantification of CD11b^+^Gr-1^+^ cells in the tumor.

**Figure S31.**Representative flow cytometry plots of immune cells in the tumor from the MC38 subcutaneous tumor treatment model. (A) CD8^+^ T cells. (B) CD4^+^CD44^+^CD62L^+^ cells. (C) CD8^+^CD44^+^CD62L^+^ cells.

**Figure S32.** Therapeutic effects of SFE neoadjuvant vaccination in the TC-1 tumor metastasis and recurrence model. (A) Mouse body weight. (B) Mouse tumor weight. (C) Mouse spleen weight.

**Table S1.** The list of antibodies used in flow cytometry in this study.

| Antibody Name | Channel | Species | Manufacturer | Catalog Number |
| --- | --- | --- | --- | --- |
| CD3 | Pcy5.5 | M | Elabscience | F1013J |
| CD4 | FITC | M | Elabscience | F1097C |
| CD8a | PE | M | Elabscience | F1104D |
| CD11b | FITC | M | Elabscience | F1081C |
| Gr-1 | PE | M | Elabscience | F1120D |
| IFN-γ | APC | M | Elabscience | F1101E |
| CD44 | APC | M | Elabscience | F1100E |
| Foxp3 | PE | M | Elabscience | F1238D |
| CD62L | Pcy5.5 | M | Elabscience | F1011J |
